# ‘Having skin in the game’: guiding principles for incorporating moulage into OSCEs

**DOI:** 10.1186/s41077-024-00307-1

**Published:** 2024-08-15

**Authors:** Bilal Korimbocus, Helen Wilson, Aine McGuckin, Gerard J. Gormley

**Affiliations:** 1 Northern Ireland Medical and Dental Agency, Belfast, UK; 2 Centre for Medical Education, Queen’s University Belfast, Belfast, UK; 3 School of Pharmacy, Queen’s University Belfast, Belfast, UK; 4 Simulation, InterSim’s University Belfast, Belfast, Queen UK

**Keywords:** Moulage, Dermatology, OSCE, Simulation

## Abstract

**Background:**

Dermatological conditions are a common reason for patients to seek healthcare advice. However, they are often under-represented in Objective Structured Clinical Examinations (OSCEs). Given the visual nature of skin conditions, simulation is suited to recreate such skin conditions in assessments such as OSCEs. One such technique often used in simulation is moulage—the art and science of using special effects make-up techniques to replicate a wide range of conditions on Simulated Participants or manikins. However, the contextual nature of OSCEs places additional challenges compared to using moulage in more general forms of simulated-based education.

**Main body:**

OSCEs are high-stakes assessments and require standardisation across multiple OSCE circuits. In addition, OSCEs tend to have large numbers of candidates, so moulage needs to be durable in this context. Given the need to expand the use of moulage in OSCE stations and the unique challenges that occur in OSCEs, there is a requirement to have guiding principles to inform their use and development.

**Conclusion:**

Informed by evidence, and grounded in experience, this article aims to provide practical tips for health profession education faculty on how best to optimise the use of moulage in OSCEs. We will describe the process of designing an OSCE station, with a focus on including moulage. Secondly, we will provide a series of important practice points to use moulage in OSCEs—and encourage readers to integrate them into their day-to-day practice.

## Background

Objective Structured Clinical Examinations (OSCEs) are a dominant form of assessment in health professional education. Since their origin, nearly 50 years ago, OSCEs have facilitated the assessment of interactional and behavioural skills [1]. The content of OSCEs is determined by a ‘blueprinting’ process, where outcomes of a course are systematically sampled and represented in an OSCE [2]. It is important that all conditions have equal opportunity to be included. Real patients, with actual clinical features, may be incorporated into an OSCE, but there are challenges in integrating such individuals into this form of assessment [3]. For instance, the need to standardise clinical signs across different OSCE circuits—in order to achieve an equitable assessment experience for all. Additionally, it may be inappropriate to allow large cohorts of candidates to examine real patients repeatedly (e.g. examination of a patient with painful leg cellulitis) or due to the nature, and sensitivity, of the illness (e.g. assessing a patient with a potential melanoma). The design and delivery of OSCE stations often draw upon the principles of simulation to create pseudo-clinical encounters for the purposes of assessment. This includes taking a scripted history from a simulated participant (SP), conducting a procedure on a part-task manikin (e.g. venepuncture on a manikin arm), hybrid simulation (e.g. suturing a ‘wound’ on a suture pad that is attached to an SP’s arm) or by using a full body manikin (e.g. managing a ‘patient’ who is having a cardiac arrest).

Despite the high prevalence rate of dermatological conditions in our populations and a common reason to consult with a family physician, they are often under-represented in medical assessments [4]. Therefore, it is important we strive to include dermatological conditions in our health profession assessments. Given the visual nature of dermatological conditions and manifestations of disease, simulation is well-placed to recreate such skin conditions. One such technique that is used widely in simulation is *moulage*. Moulage is a term derived from the French term *moule* (‘to mold’) which describes the process of using special effects make-up techniques to replicate a wide range of conditions [5]. Often applied to SPs, moulage can take the form of make-up applied to the skin (e.g. bruising), the application of moulds to the skin (e.g. a skin wound), temporary transfer tattoos (e.g. a transfer melanoma tattoo) or a mixture of all these techniques [5–8]. In the context of an OSCE station, such dermatological moulage aims to provide important visual clues that are relevant to the assessment objectives. However, the contextual nature of OSCEs places additional challenges compared to using moulage in more traditional forms of simulated-based teaching situations. Summative OSCEs are high stakes and require a high degree of standardisation across multiple OSCE circuits [9]. Moreover, OSCEs tend to have large numbers of candidates, so moulage needs to be robust and durable. Given the need to expand the use of moulage in OSCEs and their unique challenges, there is a requirement to have guiding principles to inform their use and development. This article aims to provide practical tips on how best to optimise the use of moulage in OSCEs. We draw upon evidence, but importantly the experience of practitioners in the field of simulation – in sharing practical insights that are grounded in the real-life practice of using moulage in OSCEs. Firstly, to understand how to include moulage into an OSCE station, we will provide a broad overview of the essential steps in designing an OSCE station, with a focus on how this relates to including moulage. Secondly, we will provide a series of important practice points to optimise the use of moulage in OSCEs. It is our hope that such practical tips could be included in faculty training programmes and policy documents for creating OSCEs. Moreover, many of these tips could also be applied to the use of moulage in more general forms of simulation-based education.

### The design process of incorporating moulage into an OSCE station

**Fig. 1:**
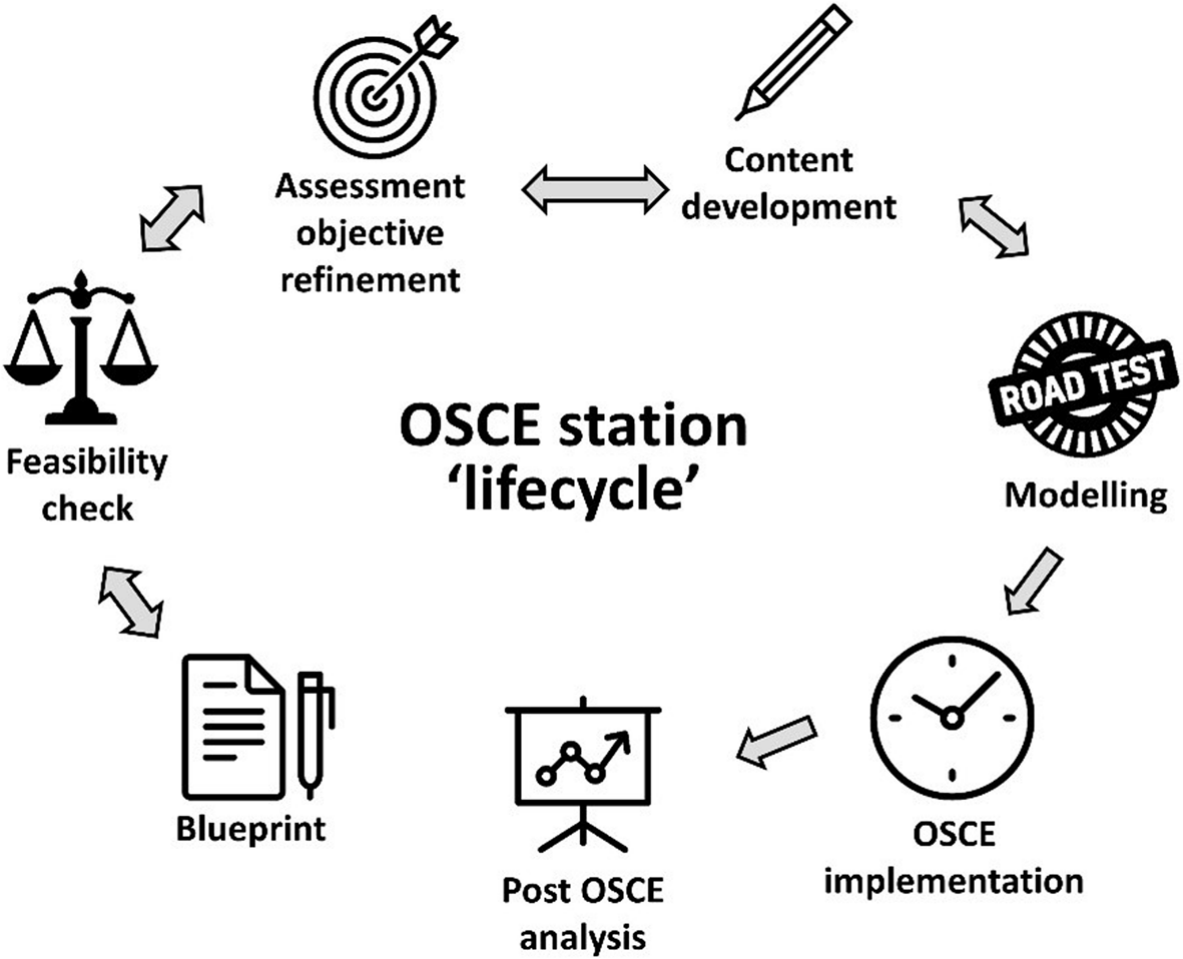
Illustration of the design process, and delivery, of an OSCE station

The design and delivery of high-standard OSCEs is an important remit in health profession curricula. Often it is an iterative process that involves careful planning, piloting, delivery and post-OSCE analysis to refine the station for future use, including its psychometric properties [10]. Figure 1 illustrates the ‘lifecycle’ of developing an OSCE station.

If a dermatological condition is to be considered in an OSCE, it is important that they are included in the blueprinting process. Often at this stage, dermatological conditions in an OSCE may be dismissed due to perceived practical challenges. However, with the advancement of simulation and moulage techniques, this may not be the case. If a dermatological condition is to be included in the OSCE, it is prudent that the feasibility of this is determined at an early stage, before much effort is invested in incorporating this condition into an OSCE. Seeking advice from those who have experience in creating moulage design is critical at this juncture (see later for the importance of having the input of a multiprofessional team). If the moulage is deemed feasible, then it is important to refine the OSCE assessment objective. This is of the utmost importance, as all efforts in crafting an OSCE station will be based on this. For example, is the assessment objective taking a history and examination from a patient who has a suspicious skin mole? Or is it to breaking bad news to a patient who may have a melanoma?

Once the assessment objective has been determined, the drafting of the OSCE station takes place. This includes the candidate question, marking scheme, SP guidance, examiner instructions and any technical set-up required for the OSCE station. As ever, we would always advocate the involvement of individuals with lived experience of illness to help co-produce such OSCE stations (for example, an individual who lives with psoriasis) [11]. Following this, it is important to ‘road test’ the OSCE station before the actual assessment day. This will help to mitigate and identify any issues before the OSCE station goes live (especially in the context of a high-stakes OSCE). Once refinements have been made, the station is ready to be delivered. If possible, having the OSCE station writer present during the OSCE helps address any interpretive issues from either examiners, SPs or indeed candidates. Following the OSCE, feedback should be sought from all those that took part in the OSCE. This information will be invaluable in modifying and improving the station for future use. As ever, the insights from a psychometrician will also help guide the enhancement of the station.

### Top tips for incorporating moulage into an OSCE station

Given the nature and requirements of moulage in OSCEs, there are some practice points that are worth considering in optimising their use and integrating into the ‘life cycle’ of an OSCE station. Below we will describe a series of important recommendations to enhance the success of using moulage in OSCEs.

#### Tip 1: ‘Wisdom of the crowd’: a multi-disciplinary team effort

**Fig. 2:**
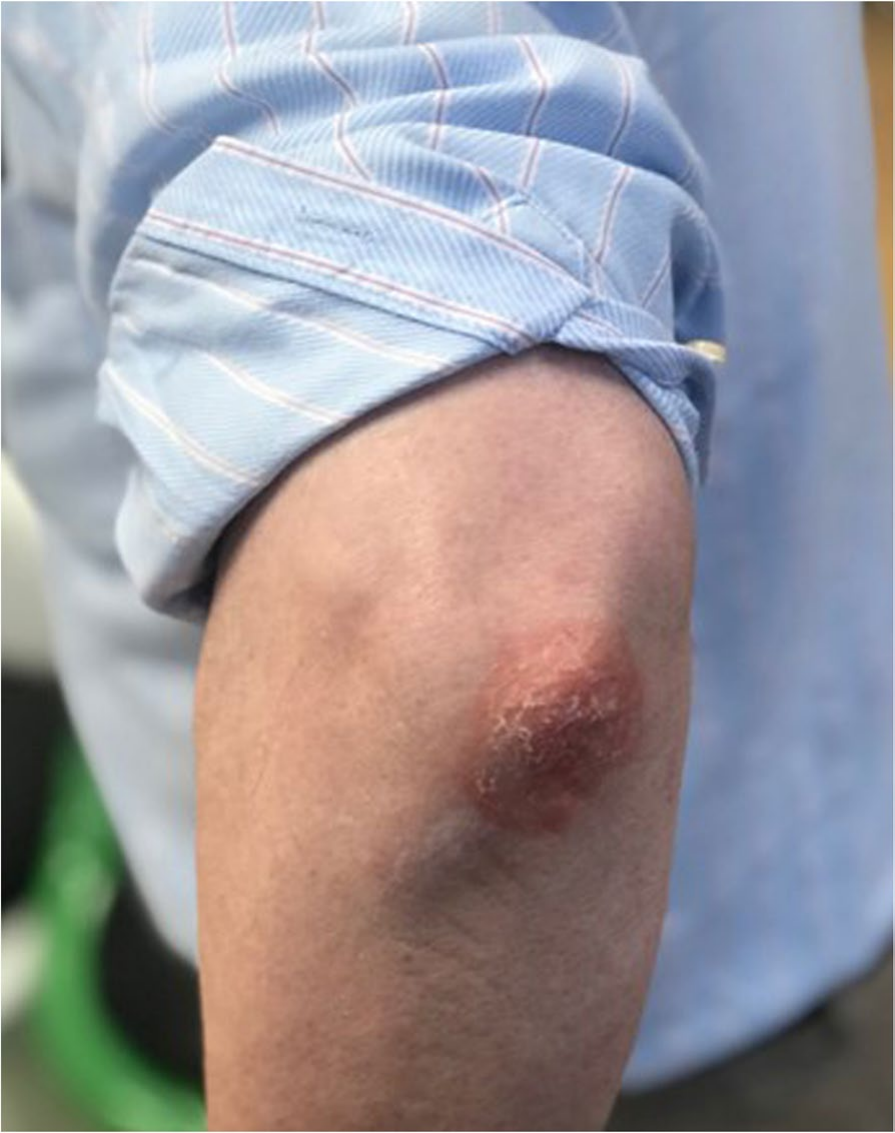
Moulage of plaque psoriasis

As in clinical practice, having a multidisciplinary team will ensure the effective use of moulage in an OSCE station. Personnel to consider are individuals who have moulage skills, academics, healthcare professionals and SPs. Critical to the process is calling upon the skills of an individual who has experience in moulage. This is not only in applying moulage, but they may also help to develop new types of moulage that were previously not thought possible. For example, in this moulage of plaque psoriasis, we were able to work with a special effects makeup artist who provided guidance on how to achieve the red base colour and skin scales using silicone (see Fig. 2). Not all units may have experts in moulage (e.g. special effects makeup artists). Therefore, it is worth considering a staff member (e.g. a simulation technician) to be trained in the skills of moulage (e.g. special effects makeup course). As ever, a healthcare professional will provide invaluable advice about the nature of the skin lesion (see “Tip 2: ‘Genuine fake’: ensuring clinical authenticity” section) and often SPs can offer insights into the usability and practical aspects of running the moulage in an OSCE.

#### Tip 2: ‘Genuine fake’: ensuring clinical authenticity

**Fig. 3:**
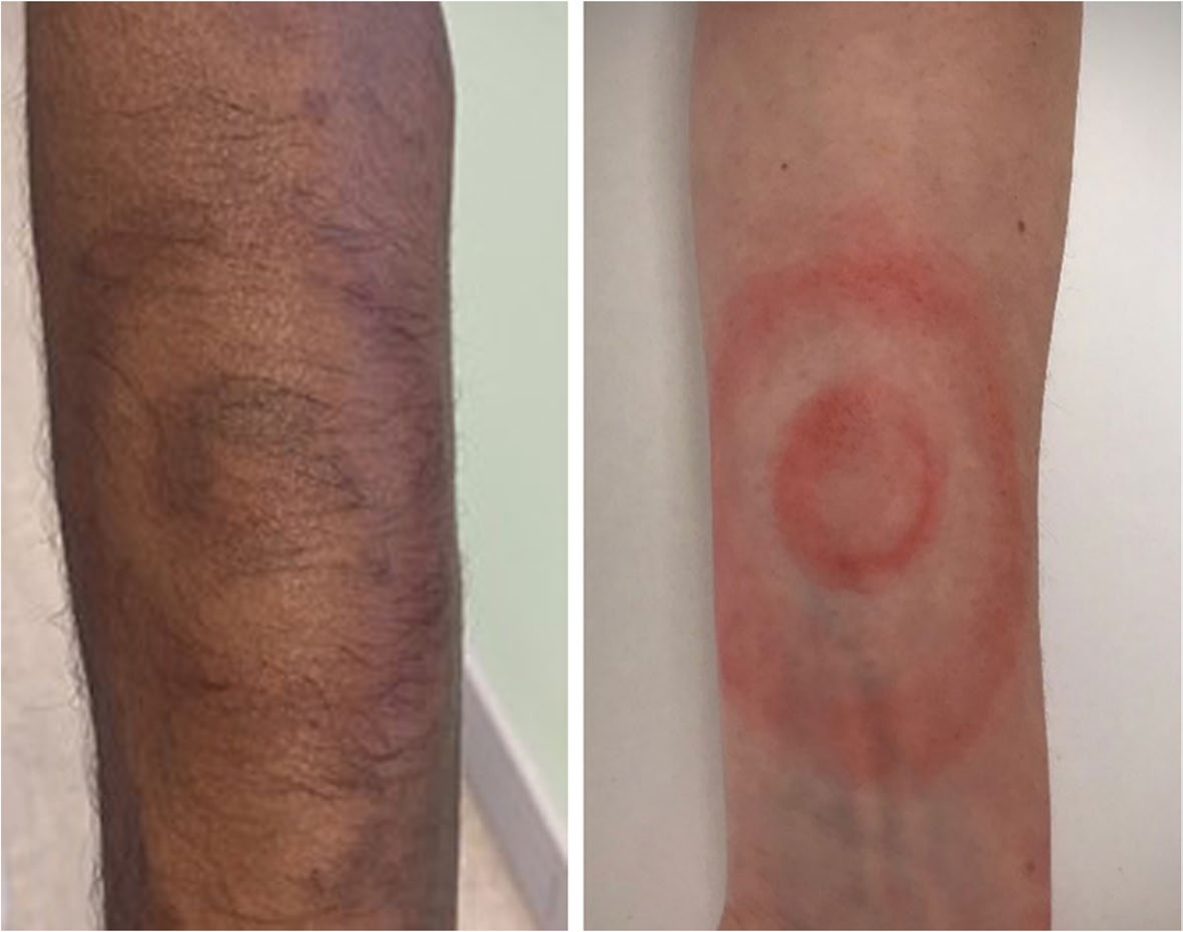
Moulage of an erythema migrans skin rash

Given that candidates may be judged on their ability to diagnose a dermatological condition, the moulage must be as authentic as possible and enable candidates to be immersed in the OSCE station [12], not just in terms of aesthetics but also the morphology of the skin condition. Having such physical realism is important in enabling candidates to suspend disbelief that the dermatological condition that is being simulated is as close to real life as possible [6, 12]. Therefore, it is prudent to have a clinical specialist involved in the process to ensure that the features of the moulage align with the actual skin condition. For example, in this moulage we wanted to create the rash associated with Lyme disease (i.e. Erythema Migrans or ‘Bullseye’ rash, see Fig. 3). In devising such a moulage, the advice of a clinician, such as a dermatologist or family physician is invaluable. In addition to the visual morphology that is represented in the moulage, other supporting features are also important in the OSCE station (for example, in the SP script, noting that they have been recently bitten by a tick; the rash is expanding and often (but not exclusively) the rash is present in the leg area) [13].

#### Tip 3: Durability of the moulage in an OSCE

**Fig. 4:**
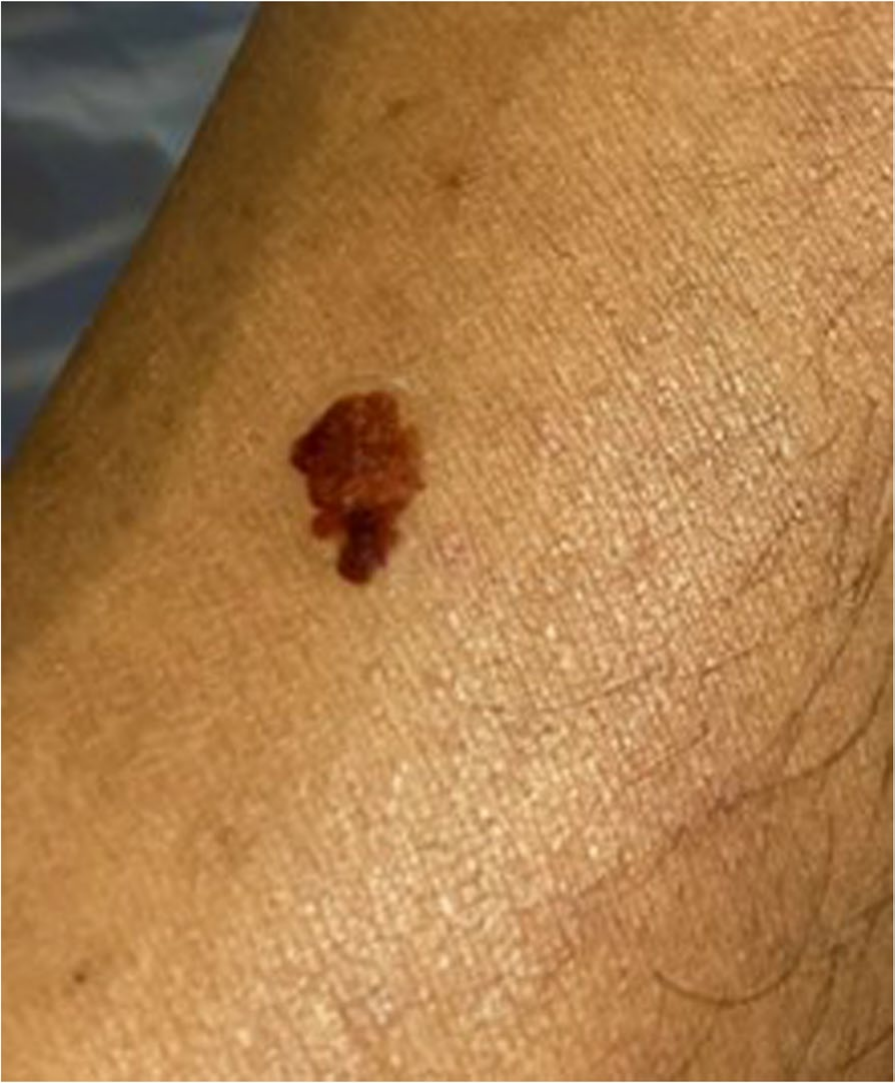
Transfer tattoo of a melanoma

Often OSCEs will have large cohorts of candidates. Furthermore, candidates may be required to physically examine and touch the moulage. Given the need to afford all candidates the same assessment experience, it is important that the moulage is durable throughout the OSCE process. For example, there are some skin paints that are more durable than others, including moulage paints that are activated by alcohol. From our experience, transfer skin transfer tattoos are durable in OSCEs [7, 8]. They have withstood multiple examinations and are easily removed with soapy water at the end of an OSCE (see Fig. 4). It is always worth considering applying more than one moulage, so the SP can easily revert to the other moulage if there are any issues.

#### Tip 4: Cost-effectiveness and efficiency in the application of the moulage

**Fig. 5:**
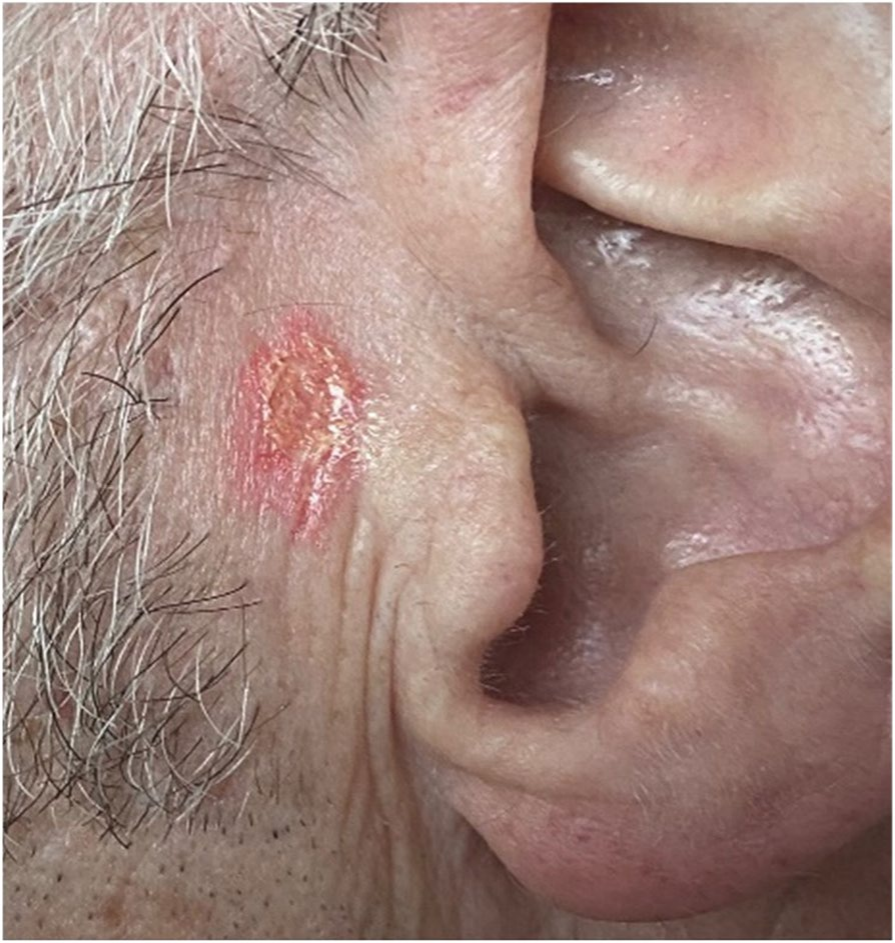
Moulage of a squamous cell carcinoma

OSCEs are busy and time-critical events. Therefore, the application of moulage should be as easy and efficient a process as possible. Often it is prudent to have more than one individual who can apply the moulage, especially as there may be several SPs, across several OSCE circuits. Asking your SPs to attend in good time, ahead of the OSCE start time, is important. Moreover, elaborate moulages that can take experts significant time to create (e.g. a complex wound) may not be possible in an OSCE. In this example, the moulage is aimed to create a squamous cell carcinoma (see Fig. 5). Applying the base colours and the raised surface using silicone paste took a non-expert in moulage 5 min to create. The melanoma transfer tattoo (Fig. 4) takes less than 1 min to apply [7]. It is important to consider the costs of moulage [14], particularly on the large-scale nature of OSCEs. Moulage can be expensive, therefore it is important to consider and identify adequate funding to support the use of moulage in OSCEs. Interestingly, the moulage used in the images in this article comes at a low material cost. For example, the material costs for the melanoma transfer tattoos in Fig. 4 cost < £0.01/$0.01/€0.01 per tattoo [7].

#### Tip 5: Be inclusive and not exclusive

With an ever-growing movement to promote Equality, Diversity and Inclusion (EDI) and decolonise the health profession curricula, moulage provides an excellent opportunity to review and evaluate how we assess various dermatological conditions [15, 16]. When designing moulage for OCSEs, it is important to consider how the ‘real life’ lesion presents on various skin tones and reflect this on the moulage that is created. The MDT should consider how the skin condition chosen may present across all skin tones and ensure this is evidence-based and guided by your team. For example, the melanoma tattoo above is a histologically true melanoma lesion that was photographed using a high-resolution camera. This is accurate and representative of how this cancer would present on different skin tones and thus this temporary tattoo can be applied to SPs with a range of skin tones. However, it may not always be possible to use the same moulage on different skin tones. For example, the images of Erythema Mirgrans (Fig. 3) required different skin tone palettes to achieve different looks. Therefore, it is important to ensure the necessary equipment (e.g. make-up palettes) is sourced to cover the spectrum of skin tones.

#### Tip 6: No one should be hurt: SP safety

Safety of the SPs, and candidates, is paramount when using moulage in OSCEs, not just in terms of physical safety but also psychological, emotional and cultural safety [17–19]. Make sure to only ever use products that are safety tested and approved for human use. Always consider the risk of skin allergies and products that you are using and determine if the SP has any allergies. For example, some moulage products contain latex and some individuals may have an allergy to this material. We would recommend performing a risk assessment and having good governance structures in place for the use of moulage in OSCEs. Equally, if candidates are expected to touch the moulage (e.g. with ungloved hands)—it is worth checking if any candidates have a skin allergy and taking any necessary steps to avoid exposure to such triggering allergens.

**Fig. 6:**
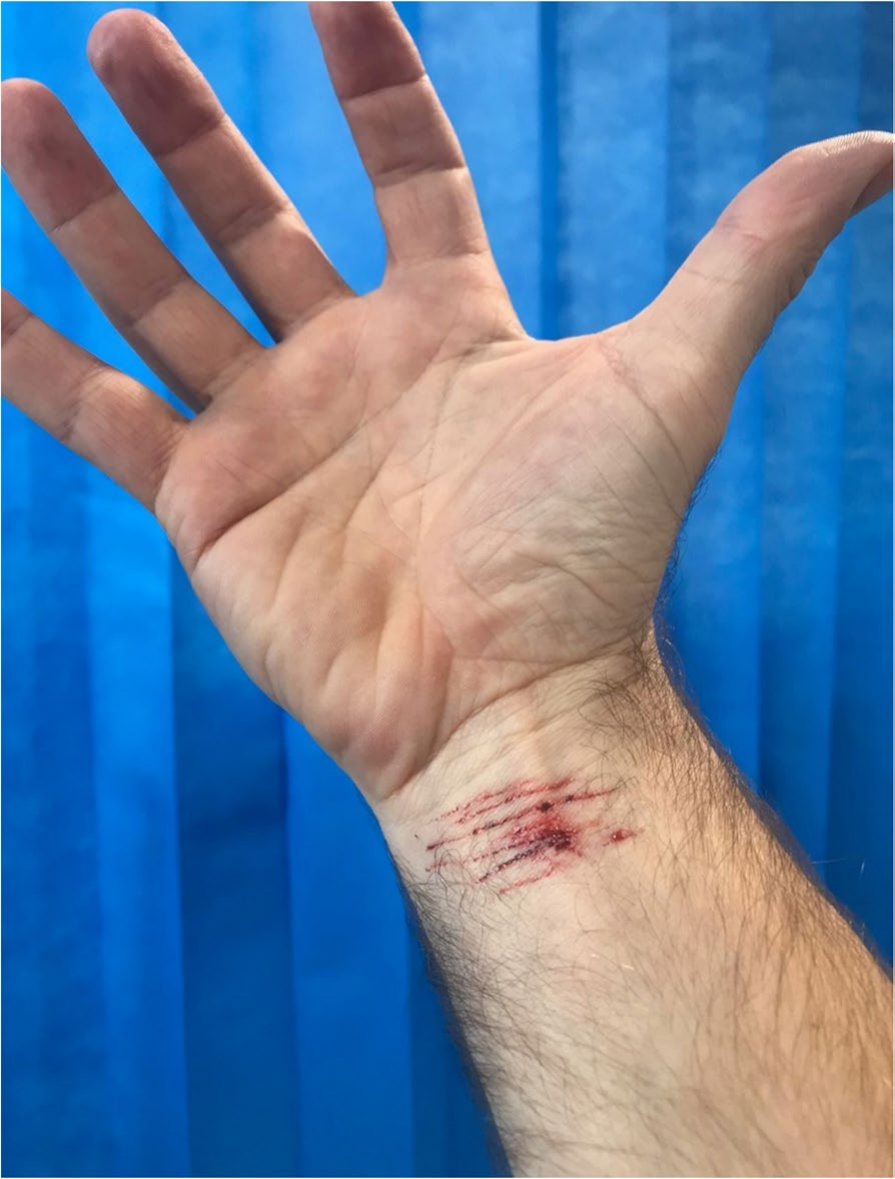
Transfer tattoo of superficial skin lacerations

We also recommend considering the psychological, emotional and cultural safety of SPs when using moulage. Given the high degree of realism that can be created using skin moulage, this can potentially be traumatising for an SP. For example, the use of a melanoma tattoo or self-inflicted skin lacerations could be a psychological trigger for some individuals causing a re-traumatising experience (see Fig. 6). Be attentive at all times and consider trauma-informed approaches when using moulage [17]. For example, conducting a fully informed consent process with an SP, with the option to withdraw at any stage. Following the OSCE, SPs should be debriefed and offered any support if they have any ongoing issues.

## Conclusion

In this article, we set out to provide practical tips on the use of moulage in OSCEs. This has been informed by evidence and grounded in experience. Given the importance of skin conditions, simulation principles have much to offer in helping the optimal use of moulage in OSCEs. OSCEs have important contextual factors that provide additional challenges compared to traditional simulation-based teaching. By means of these practical tips, we would encourage practitioners to use and expand the range of moulage in OSCEs. Beyond assessment in OSCEs, many of the principles offered in this article could also guide the use of skin moulage in teaching contexts (e.g. teaching large cohorts of medical and nursing students). More than just teaching about knowledge and skills, moulage has the potential to enhance skills such as empathy [20, 21]. With the greater use of moulage in health profession education, many have called for guiding frameworks to pedagogically leverage its use [20]. Therefore, our practical tips could be included in faculty training programs and policy documents for creating teaching encounters and OSCEs that utilise skin moulage. Through careful planning, with a multi-disciplinary team, with particular attention to the durability, authenticity and safe use of moulage, we hope this guidance has been helpful in allowing skin conditions to be represented in OSCEs. Critically, such moulage needs to be inclusive and represent our diverse population. Finally, we are aware that assessment has the potential to drive learning [22]. With more dermatological conditions in OSCEs, the use of moulage may have an unintentional consequence of steering learning about dermatological conditions.

## Data Availability

Not relevant to this descriptive manuscript.

## Abbreviations

OSCEs: Objective Structured Clinical Examinations
SPs: Stimulated participants
